# A new Eastern Asian *Hycleus* and key to the Chinese species of the *phaleratus* group (Coleoptera, Meloidae, Mylabrini)

**DOI:** 10.3897/zookeys.463.8261

**Published:** 2014-12-12

**Authors:** Zhao Pan, Monica Carosi, Marco A. Bologna

**Affiliations:** 1 The Key laboratory of Invertebrate Systematics and Application of Hebei Province, Hebei University, 071002, Baoding, Hebei Province, China; 2 Dipartimento di Scienze, Università degli studi Roma Tre, Viale G. Marconi 446, 00146, Rome, Italy

**Keywords:** Blister beetles, new species, China, key to species, taxonomy

## Abstract

A new species of *Hycleus* belonging to the *phaleratus* group, and close to *Hycleus phaleratus*, is described. The new species, *Hycleus marcipoli*, is distributed in China (Gansu and Taiwan), Laos, and northern Thailand. A key to the Chinese species of this group is presented.

## Introduction

*Hycleus* Latreille, 1817, tribe Mylabrini, is the most speciose genus of the blister beetle family with approximately 430 described species. However, the only study of the genus is a very old comprehensive one (Marseul 1872) devoted to the entire tribe. *Hycleus* itself remains inadequately studied and is in need of complete revision. The genus is restricted to the Old World and centered in the Afrotropical region (Bologna and Pinto 2002). In the literature it has been confused with the genus *Mylabris* Fabricius, 1775 and other Mylabrini genera by several Authors. The very complex synonymy and generic definition of *Hycleus* were explained by Bologna (1978, 1991) and Bologna and Pinto (2002).

The taxonomy of some Palaearctic and Afrotropical species groups have been studied in the last 50 years (e.g. Pardo Alcaide 1954, 1955, 1958, 1968; Bologna 1978, 1979, 1990, 1991), while that of the Oriental species is still confused and the scanty contributions provide rough descriptions and figures (Saha 1972, 1979). Recently, a preliminary taxonomic study of Chinese *Hycleus* species was published by Pan et al. (2011a, in Chinese); this contribution considered 19 species belonging to both Palaearctic and Oriental lineages. As is widely known, the Chinese area belongs to both biogeographic regions and is also characterized by a transitional biogeographic subregion in its south-eastern portion (e.g., Palestrini et al. 1987; Brown and Lomolino 1998) thus representing a valuable and appropriate research zone.

Several Oriental species of *Hycleus* belong to the *phaleratus* group, which is widely distributed from Pakistan to eastern China and Indonesia. The *phaleratus* group is distinct, but more closely related to Afrotropical than to Palaearctic lineages. It is mostly distributed in the Oriental region and in the transitional biogeographic subregion, and marginally spread in some Palaearctic zones of Mongolia, China, Himalayan countries, India and Pakistan. This group of species belongs to the *Hycleus* lineage characterized by a mesosternum of the Mesoscutatus type (see Bologna 1991) with a large modified fore area (“scutum”). It is easily distinguishable from other *Hycleus* belonging to the Mesoscutatus lineage by the following characters: male maxillary galeae not distinctly modified; antennae with 11 antennomeres entirely black (Fig. 3); elytra black with two reddish yellow basal spots (one in the middle and one on the external margin) and two, middle and subapical, yellow-reddish transverse fasciae (Fig. 4).

The species of this group have been repeatedly confused in the literature and the future examination of types will be the basis for a taxonomic revision of all included species. The main taxonomic problem is that Pallas (1782) described “*Meloe phalerata*” from “*cisgangeticae Indiae*” (type locality: an old name indicating the Indian regions W of Ganges river), but its collection is missing and no types are currently available. The identification of *phaleratus* in the literature is doubtful and has always been based on the elytral pattern, which actually is similar in other Oriental species of this group. Considering that this taxonomic problem remains unclarified, in the present paper we defined as *phaleratus* the species occurring in India; it best corresponds to the Pallas’ description.

Working on Chinese specimens of this group housed in the M. Bologna’s collection (University Roma Tre: MAB), we discovered a new species not identified in the Pan et al. (2011a, b) studies. Aim of the present study is to describe this new species and to provide a key to the known Chinese species of the *phaleratus* group.

## Results

### Hycleus marcipoli

Taxon classificationAnimaliaColeopteraMeloidae

Pan & Bologna sp. n.

http://zoobank.org/1EA7C534-A836-4854-A110-403CD7CFA9CC

Figures 1a , 2–9

#### Type specimens.

Holotype male (MAB), labelled “China, Kansu mer. Shinlong-Shan Mts. Yuzhong, 3200 m a.s.l., 6/7.VII.1998, L. Bieber leg.” (white, rectangular, printed). **Paratypes:** 3 females (MAB) with the same label of holotype; 1 female (MAB), labelled “Formosa” (white, rectangular, printed); 1 male (MAB), labelled “Thailandia, Ghiang Dao, Chiang Mai” (white, rectangular, printed); 1 female (MAB) labelled “Nord Thailand, Doi Chiang Dao, 1300 m, 20.IX.1979, T. Racheli leg.” (white, rectangular, printed); 1 female (MAB), labelled “Laos, Luang Prabang, 20.VII.1975, Rossetto leg.” (white, rectangular, printed); 2 males and 1 female (MAB), labelled “Laos, Vientiane, Phu Khao Khoay, 15.V.2006, D. Macale leg.” (white, rectangular, printed); 6 males and 7 females (MAB) labelled “Laos, Oudomxay prov., Namo distr., Phouxang, 10-26.VI.2008” (white, rectangular, printed). All types have additional labels “Holotypus (and Paratypus, respectively), *Hycleus marcipoli* sp. n. Z. Pan & M. Bologna det. 2014” (red, rectangular, printed and handwritten).

#### Type locality.

“China, Kansu mer. Shinlong-Shan Mts. Yuzhong”. Shinlong-Shan Mts., as written on the label, is the transliterated name of the Xinglong-Shan Mts., located in the Yuzhong County, Lanzhou City, in Southeastern part of Gansu Province. These mountains represent the eastward extension of the Qilian-Shan Mts. This area is usually included in the Palaearctic region, but according to its animal and plant diversity, it belongs to the transitional Chinese area.

#### Diagnosis.

**Figure 1.**
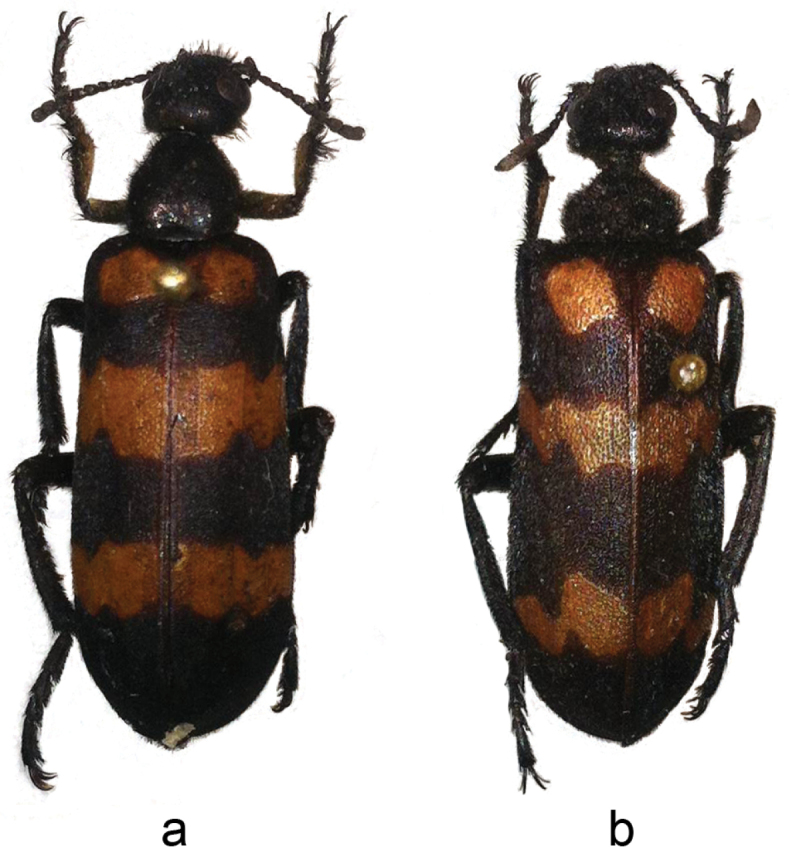
Habitus, male, in dorsal view, **a** *Hycleus marcipoli*, holotype **b** *Hycleus biundulatus*.

This is a species of the Mesoscutatus type lineage, belonging to the *phaleratus* group and phenetically similar to *Hycleus phaleratus* (Pallas, 1782). Body only with black setae except the elytral axillary fore spot, the protibiae and protarsi with mixed black and yellow-brown setae. Basal part of antennomere XI narrower than the apical part of antennomere X (Fig. 3). Elytral yellow-reddish fasciae wider and slightly flexuous (Figs 1a, 4). Fore margins of mesepisterna forming a median narrow groove, margins almost touching each other (Fig. 5). Apical setae on external side of protibiae distinctly longer than those on other parts of tibia, reaching the apical margin of protarsomere I. Proximal aedeagal dorsal hook positioned far from the distal one (Fig. 6).

#### Description.

Body (Fig. 1a) unicolour black, except elytra, which are black but with two testaceous fore spots (one axillary and one close to the scutellum), two slightly sinuate testaceous fasciae, a medial one and a subapical one (Fig. 4). Body with black setae, also on ventral side, but mixed with golden setae on the elytral axillary fore spot, on inner side of protibiae, and on protarsal pads. Body length (apex of mandibles-apex of elytra): 26–38 mm.

**Figures 2–9.**
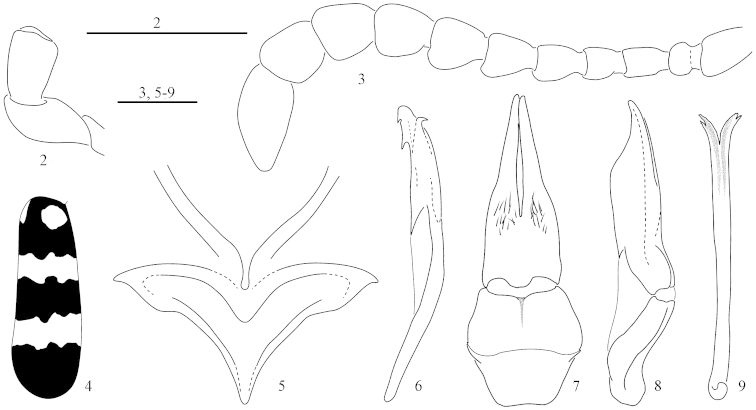
*Hycleus marcipoli* sp. n. **2** male labial palpi, in ventral view **3** antenna **4** elytral pattern **5** mesosternum and mesepisterna **6** aedeagus, in lateral view **7** tegmen, in ventral view **8** tegmen, in lateral view **9** *spiculum gastrale*. Bar scales: 1 mm.

Head subquadrate, slightly longer than wide, with the maximum width at the level of eyes. Punctures shallow, medium in size and quite dense, in some specimens reduced near the frontal suture, with an inconspicuous depression in the middle, between eyes. Eye globose, with the antero-dorsal margin slightly sinuate, just behind the antennal insertion. Temple subparallel, only slightly curved posteriad and subequal in length to the longitudinal diameter of eye. Clypeus, narrower than the interocular width, rounded on sides, posteriorly with same punctures of frons and anteriorly almost smooth and slightly sloping; labrum subreniform, scarcely narrower than clypeus, rounded on sides, the fore margin moderately sinuate in both sexes, medially slightly depressed. Male maxillary galeae non modified in both sexes, laterally and ventrally, with a tuft of elongate robust setae, not thickened, on posterior half; maxillary palpomeres slightly enlarged apically, particularly II and III, palpomere IV suboval; labial palpomere II slightly widening (Fig. 2). Mandibles curved and progressively narrowed on the apical third. Antennae with 11 antennomeres (Figs 3): I-V more or less shiny, the remaining subopaque; antennomere I ca. as long as II-III together; II subglobose; III-IV subcylindrical and slender, III about 1.5 times as long as IV; V-VIII similar in length, subtrapezoidal, apically enlarged on external side, increasing in width from V to IX and then decreasing from X to XI, X subquadrate and slightly shorter than IX; antennomere XI distinctly narrower and ca 1.5 as long as X, subcylindrical but narrowed in the apical third.

Pronotum elongated, distinctly longer than wide, about as wide as head at eyes, subparallel on sides on the basal 2/3, and then distinctly narrowing anteriad; fore portion greatly depressed, as well as on the middle of base, just in front of mesonotum; punctures similar to that on head, with a longitudinal medial small furrow, almost impunctate. Elytral pattern as in Figs 1a, 4. Mesosternum of the Mesoscutatus-type (Fig. 5); fore margins of mesepisterna forming a median narrow groove, margins almost touching each other. Legs slender; protibiae with two spurs, both tibial spurs on all legs slender; protarsi in both sexes with a distinct golden ventral pad; apical setae on external side of male distinctly longer than those on other parts of the tibia, reaching the apical margin of protarsomere I; external side of male protibiae with scattered longer setae, and inner side on both sexes with dense golden setation. Protarsi as long as protibiae, protarsomeres always longer than wide, slightly widened apically and with dense and longer setae at apex.

Posterior margin of the penultimate male abdominal sternite sublinear, that of the last visible sternite only slightly emarginated. Parameres (Figs 7, 8) distinctly elongate with slender and elongate apical lobes, much narrower than basal third in ventral view; aedeagus with two slender hooks, proximal one positioned far from the distal one (Fig. 6); endophallic hook small and slender.

#### Etymology.

As a tribute to the collaboration established among the authors during the Ph.D. studies made in Italy by one of them (PZ), the new species is named after Marco Polo (1254–1324), the Venetian explorer who, during a long period of permanence in China in the late XIII century (1271–1284), established the first well documented relationships between the Chinese and European worlds and opened western culture to the wide and rich Chinese heritage.

#### Taxonomic remarks.

The species of the *phaleratus* group are phenetically similar in body shape, but variable in size and elytral colouration; *Hycleus phaleratus* is the most similar to *Hycleus marcipoli*. These species have been repeatedly confused due to their similar, but they can be identified by the following characters used in the key below: colour of setae on ventral side of body and on elytral yellow-reddish fasciae and spots, especially the axillary spot; length of setae on male protibiae and protarsi; shape of protarsi, mesosternum, and male genitalia; extension of the fore pronotal depression.

#### Distribution.

China (SE Gansu, Taiwan); Laos; Thailand.

### Key to the Chinese *Hycleus* species of the *phaleratus* group

**Table:** 

1	Male protibiae with two apical spurs	**2**
1’	Male protibiae with one apical spur only. China (Fujian, Hubei, Sichuan, Yunnan)	***hirtus* (Tan, 1992)** (the validity of this species needs to be confirmed)
2	Setae black, except a mixture of black and golden setae on elytra, tarsi and protibiae	**3**
2’	Body setae mixed golden and black, at least on the ventral side of thorax, and possibly on other parts of body	**5**
3	Elytral reddish-yellow fasciae narrow, distinctly flexuous and jagged (Fig. 1b). China (Fujian, Yunnan); Indonesia (Java); India; Sri Lanka; Pakistan	***biundulatus* (Pallas, 1782)** (syn. *pustulatus* Thunberg, 1791)
3’	Elytral reddish-yellow fasciae wider and slightly flexuous, not jagged	**4**
4	Elytral axillary spot with few yellow setae mixed to black setae; setae at external apex of male protibiae longer than those on other sides and reaching the apical margin of protarsomere I; fore margins of mesepisterna forming a median narrow and drop-like groove, margins almost touching each other; proximal aedeagal hook positioned far from the distal one. (Figs 1a and 2–9). China (SE Gansu, Taiwan); Laos; Thailand	***marcipoli* Pan & Bologna sp. n.**
4’	Elytral axillary spot with black setae only; setae at external apex of male protibiae longer than that on other parts, not reaching the apical margin of protarsomere I; fore margins of mesepisterna almost parallel along the median groove and posteriorly diverging, median groove wide and almost parallel; proximal aedeagal hook close to the distal one. SE China; Thailand; Indonesia (E to Timor Is.); Nepal; India; Sri Lanka; Pakistan (also in its Palaearctic part) (the true distribution must be better defined)	***phaleratus* (Pallas, 1782)**
5	Elytral yellow-reddish fasciae with mixed yellow and black setae, but the axillary spot	**6**
5’	Elytral yellow-reddish fasciae with black setae only, but the axillary spot	**7**
6	Body small to middle sized (length 11.9–21.7 mm); eyes normal in size, ca. 0.6 as long as head; antennomere XI more than 1.5 as long as wide; proximal aedeagal hook relatively far from the distal one (Fig. 5J, in Pan et al. 2011a). SE China (Guangxi, Yunnan, Guangdong, Taiwan, Hong Kong); Vietnam; Laos; Cambodia; Thailand; Indonesia (Java); Nepal; Sikkim; N India. Doubtfully recorded from Japan	***cichorii* (Linnaeus, 1758)**
6’	Body size small (length 10.0–13.3 mm); eyes longer, ca. 0.75 as long as head; antennomere XI distinctly less than 1.5 as long as wide; proximal aedeagal hook relatively close to the distal one (Fig. 9J, in Pan et al. 2011a). China (Yunnan); Vietnam	***parvulus* (Frivaldszky, 1892)**
7	Protarsi short, especially in male, length of protarsomeres II-IV distinctly less than width. China (Fujian, Guangxi, Hainan, Yunnan, Taiwan, Hong Kong); Vietnam; Laos; Thailand; Myanmar; Sikkim; Nepal; N India	***brevetarsalis* (Kaszab, 1960)**
7’	Protarsi normal in length, protarsomeres II-IV longer than wide	**8**
8	Pronotal anterior depression inconspicuous; body size large, usually more than 25 mm in length; proximal aedeagal hook close to the distal one (Fig. 6J, in Pan et al. 2011a). China (Fujian, Guangxi, Sichuan, Yunnan, Xizang); Laos; Thailand; N India (Uttar Pradesh); Nepal	***dorsetiferus* Pan, Ren & Wang, 2011**
8’	Pronotal anterior depression distinct; body small to middle in size, usually less than 25 mm in length; proximal aedeagal hook relatively far from the distal one (Figs 7J, 8J, in Pan et al. 2011a)	**9**
9	Setae on dorsum of male protarsi much longer than on other surfaces; male protarsomere normally elongate and protarsomere I shorter than V; protarsi and maxillary palpi usually yellow-brown, black only in few individuals from S China; body length 14.6–24.5 mm. Mongolia; Central, Eastern and Southern China; N India (Himanchal Pradesh, Madhya Pradesh, Punjab); Nepal	***medioinsignatus* (Pic, 1909)**
9’	Setae on dorsum of male protarsi not distinctly longer than on other surfaces; male protarsomeres slender, protarsomere I as long as V; protarsi and maxillary palpi black; body length 12.3–15.6 mm. China (Sichuan, Yunnan); N India; Sikkim	***mannheimsi* (Kaszab, 1961)**

## Discussion

Most of the *Hycleus* species are distributed in the Afrotropical Region, particularly in savannah ecosystems; a large number of very distinct lineages is also spread in the Palaearctic Region, particularly in desert and steppe ecosystems. On the contrary the genus is poorly represented in the Oriental Region, probably because of the extension of primary forests, a habitat unsuitable for blister beetles.

Among the 20 *Hycleus* species distributed in China (Pan et al. 2011a, b; note that *bistillatus* (Tan, 1981), was referred erroneously to *Hycleus* but actually belongs to the genus *Mylabris*; Pan et al. in prep.), excluding *Hycleus schoennerri* (Billberg, 1813) having a Mesogorbatus type mesosternum, the remaining 19 have a Mesoscutus type meosternum. Nine of them belong to Palaearctic lineages and, in particular, the following species can be referred to the *polymorphus* group (as partially defined by Bologna 1991, 1994): *Hycleus atratus* (Pallas, 1173), *Hycleus biguttatus* (Gebler, 1811), *Hycleus chodschenticus* (Ballion, 1878), *Hycleus hokumanensis* (Kôno, 1940), *Hycleus polymorphus* (Pallas, 1771), *Hycleus quatuordecimpunctatus* (Pallas, 1781), *Hycleus scabiosae* (Olivier, 1811), *Hycleus solonicus* (Pallas, 1782), and *Hycleus tekkensis* (Heyden, 1883). The remaining ten Chinese *Hycleus* with Mesoscutatus mesosternum, here studied, belong to the Oriental *phaleratus* group.

Other species were described from India and Pakistan in the genus *Mylabris* (or its synonym *Zonabris* Harold, 1879) by Pic (1916) and Saha (1972, 1979); however, these very short descriptions and rough figures are scarcely informative. According to the descriptions and/or after type examinations, some of these can be referred to the genus *Hycleus* and a few [*ajantaensis* (Saha, 1979), *goaensis* (Saha, 1979), *gonostilus* (Saha, 1972), *himalayaensis* (Saha, 1979), *horai* (Saha, 1972), *mandibularis* (Saha, 1979), *sahai* (Kaszab, 1981) new name] may belong to the *phaleratus* group and mostly are probably synonyms of *Hycleus phaleratus*, *Hycleus biundulatus*, *Hycleus medioinsignatus* and *Hycleus cichorii*. These species apparently differ from *Hycleus marcipoli* and were never recorded from China.

The study of new characters useful in the taxonomy of Oriental lineages, such as the morphology of male genitalia, maxillae, palpomeres and mesosternal structure, utilized for the Afrotropical *Hycleus*, could support the study of Oriental species, never revised in more than one century.

## Supplementary Material

XML Treatment for Hycleus marcipoli
